# Integrated Device for Cancer Nucleic Acid Biomarker Detection at Body Temperature

**DOI:** 10.3390/mi16020192

**Published:** 2025-02-07

**Authors:** Chang Chen, Bin Wu, Xuesong Li, Yuhang Jin, Hangyu Zhang, Bo Liu, Zhengyao Zhang, Na Li

**Affiliations:** 1 Central Hospital of Dalian University of Technology, Dalian 116033, China; 2 Faculty of Medicine, Dalian University of Technology, Dalian 116024, China; 3 China Certification & Inspection Group LiaoNing Co., Ltd., Dalian 116001, China; 4 Liaoning Key Lab of Integrated Circuit and Biomedical Electronic System, Dalian University of Technology, Dalian 116024, China; 5 School of Chemical Engineering, Ocean and Life Sciences, Dalian University of Technology, Panjin 124221, China

**Keywords:** integrated device, early cancer detection, nucleic acid biomarkers, RT-RAA, colloidal gold test strip

## Abstract

The quantitative analysis of nucleic acid markers is extensively utilized in cancer detection. However, it faces significant challenges, such as the need for specialized detection devices and the inherent complexity of testing procedures. To address these issues, this study proposes a simplified, rapid, and user-friendly platform for cancer nucleic acid marker detection. We firstly designed a polydimethylsiloxane (PDMS) device for the isothermal amplification reaction of nucleic acid biomarkers based on reverse-transcription recombinase-aided amplification (RT-RAA) technology. Specifically, three potential cancer nucleic acid biomarkers, carcinoembryonic antigen (CEA), prostate-specific antigen (PSA), and prostate cancer antigen 3 (PCA3) were amplified from human serum or urine samples in the PDMS device at body temperature. The reaction chamber was directly integrated with nucleic acid test strips labeled with colloidal gold nanoparticles, allowing for the visual observation of the detection results for the amplification products. The optimal reaction conditions, such as pH, reaction time, antibody, and streptavidin concentration, were defined after a series of optimization studies. The findings demonstrated that the optimal RT-RAA reaction time was 20 min, the primary antibodies were labeled with colloidal gold to the greatest extent at pH 8.5, and the optimal concentrations of secondary antibody and streptavidin were 1.0 mg/mL and 0.5 mg/mL, respectively. Furthermore, this novel detection approach could not only exhibit excellent sensitivity and specificity but also show high accuracy for the analysis of nucleic acid biomarkers in both clinical serum and urine samples. Therefore, the simplified and more convenient operation platform provides a new insight for the semi-quantitative analysis of cancer nucleic acid biomarkers and the rapid screening of early cancer, thereby offering a promising alternative to oncological point-of-care testing (POCT) diagnostics.

## 1. Introduction

Cancer is well recognized as a severe global threat to public health and quality of life. Thus, considerable efforts have been made toward developing robust intervention and treatment strategies. However, most malignant tumors are diagnosed clinically in advanced stages because of their sneaky nature in early stages and the lack of efficacious early screening approaches, finally leading to low survival rates and the high mortality of cancer patients [1,2]. Hence, the sensitive and accurate detection of early cancer is critically imperative, especially in resource-limited regions. Current diagnostic screening techniques for tumors encompass imaging modalities, tissue biopsy analysis, biomarker identification, and so forth [3]. Tissue biopsy analysis is regarded as challenging due to its inherent invasiveness, complexity, and cost. Cancerous biomarkers are broadly believed to exhibit great capabilities in the early detection of malignant tumors, owing to their sensitivity, reliability, quantifiability, and reproducibility [4]. The quantitative analysis of cancer biomarkers from blood and urine is even regarded as the preferred diagnostic method in routine medical examination because of its advantages, such as its ease of implementation and minimal invasiveness [5].

**Figure 1:**
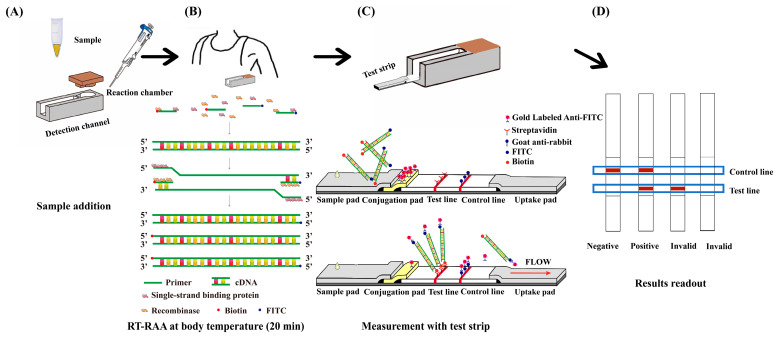
Workflow of the integrated detection platform. (**A**) Sample loading: clinical sample, primers, and reaction unit added to the chamber. (**B**) Underarm incubation: chamber incubated for 20 min for RT-RAA. (**C**) Test strip insertion: thin PDMS layer pierced to transfer reaction products to the detection channel. (**D**) Result interpretation: Negative: color only on control line. Positive: color on both control and test lines. Invalid: color only on test line.

Various biomarkers such as carcinoembryonic antigen (CEA), alpha-fetoprotein (AFP), prostate-specific antigen (PSA), and prostate cancer antigen 3 (PCA3) have been widely identified as vital indicators for early cancers [1,5,6]. These cancer biomarkers can be monitored at both the protein level and nucleic acid level [7]. Recently, nucleic acid biomarkers have demonstrated intense detection capabilities for diverse cancers at early stages [8]. The existing nucleic acid amplification assays for cancer diagnosis include quantitative polymerase chain reaction (qPCR), loop-mediated isothermal amplification (LAMP) and recombinase-aided amplification (RAA) [9]. The traditional qPCR assay has been the predominant technique for precisely and quantitatively analyzing nucleic acid due to its numerous advantages, including its rapidity, high sensitivity, specificity, and minimal sample purity requirement, but an expensive automated thermal cycler and specialized expertise are needed to perform qPCR, thereby heavily limiting its extensive application and the likelihood of point-of-care testing (POCT) [10]. Despite the low dependence of LAMP on thermocyclers, the reaction temperature is usually between 60 °C and 65 °C, necessitating temperature control equipment [11]. RAA that utilizes recombinases, single-strand binding proteins, and DNA polymerases has been performed optimally between 37 °C and 42 °C to achieve nucleic acid isothermal amplification, gradually garnering increasing attention (Figure 1B). This method can be combined with reverse transcription (RT-RAA) for detecting RNA [12,13,14,15]. This convenient novel technique enables the qualitative and sensitive analysis of nucleic acid, eliminating the need for expensive complex instrument, thus serving as an appealing alternative approach to monitor multiple nucleic acid biomarkers [16].

Although RT-RAA allows the analysis of cancer nucleic acid biomarkers in the absence of sophisticated equipment, the result still requires visualization using techniques such as agarose gel electrophoresis or fluorescent dyes by trained personnel, leading to difficulty in achieving point-of-care testing (POCT) and home self-screening. The colloidal gold immunochromatographic assay emerges as a promising solution to this problem, owing to its quick, convenient, intuitive properties [6]. These favorable features render it to be very suitable for the combination of RT-RAA technology. Based on monoclonal antibody technology, colloidal gold labeling technology, and immunochromatography, the colloidal gold test strip has been extensively employed in disease screening and diagnosis [6,17]. Furthermore, the combination of RT-RAA and the lateral flow dipstick assay has been successfully applied to detect microorganisms, such as *Helicobacter pylori* and *Candida parapsilosis* [18,19,20]. Hence, it is reasonable to speculate that the colloidal gold test strip could realize the rapid and visible detection of the RT-RAA product of cancer nucleic acid biomarkers.

To simplify the operation procedure, we established a new assay platform for detecting cancer nucleic acid biomarkers that incorporates the two kinds of technologies utilizing the isothermal characteristics of RT-RAA and the visible nature of the colloidal gold test strip. In particular, the RT-RAA reaction part and colloidal gold test strip were combined together to construct a smart integrated device that can be placed under an armpit to perform RT-RAA at body temperature followed by visualization on the test strip through unaided eyes. We hope this user-friendly and portable testing platform can facilitate the early detection of diverse cancer nucleic acid biomarkers without the requirement of sophisticated equipment and professional operators, thereby probably offering an effective practical POCT tool for rapid early cancer screening in the future.

## 2. Materials and Methods

### 2.1. Primer Design

The primers of three cancer nucleic acid biomarkers (CEA, PSA, and PCA3) for the RT-RAA reaction were designed based on sequences retrieved from the National Center for Biotechnology Information (NCBI) database (https://www.ncbi.nlm.nih.gov/, accessed on 1 May 2023). The species specificity of the primers was confirmed by utilizing the NCBI primer designing tool (https://www.ncbi.nlm.nih.gov/tools/primer-blast, accessed on 1 May 2023). To achieve visual detection, the 5′ end of the forward primer was labeled with biotin, while the 5′ end of the reverse primer was labeled with FITC (fluorescein isothiocyanate). The sequences of the primers are listed in Table 1. All the primers were synthesized by Jinkairui Biotechnology Co., Ltd. (Wuhan, China).

### 2.2. Integrated Detection Platform Design and Fabrication

The integrated detection device consisted of three parts, namely the RT-RAA reaction chamber with a cover and detection channel to house test strips separated by a thin PDMS interlayer, as well as the test strip (Figure 1). The device was fabricated using two molds that were for the reaction chamber (3.5 mm internal diameter, 5 mm height) and detection channel (19 mm length, 4 mm width), respectively, and they were printed by a 3D printer (Dreamer NX, Shanzhu Technology Co., Ltd., Zhejiang, China) using acrylonitrile butadiene styrene plastic. The two molds with a gap of 1 mm were then casted in PDMS rubber by pouring liquid PDMS (10:1 ratio of silicon base Sylgard to curing agent) in the supporting holder made of aluminum foil and tape. The liquid PDMS was cured at 80 °C in an oven for 3 h, upon which it became solidified. The PDMS device was cut into rectangular parts (30.5 mm × 10 mm). Then, the two molds were removed, leaving a cylindrical reaction chamber and a cuboid channel separated by a PDMS interlayer (1 mm thickness). At this stage, the PDMS part of the integrated device was ready for use, as shown schematically in Figure 1A. All the chemical materials were supplied by Saituo Biotechnology Co., Ltd. (Dalian, China).

After the RT-RAA reaction is completed in the reaction chamber under the armpit (Figure 1B), the thin PDMS layer is pierced with the test strip that is housed in the detection channel (Figure 1C), allowing the reaction products in chambers to enter the detection channel and be absorbed by the chromatographic strip. The whole test strip (83 mm length, 4 mm width) comprises a sample pad made of a glass fiber membrane (22 mm length), a gold-labeled pad made of a glass fiber membrane (9 mm length), a conjugate pad made of a nitrocellulose membrane (21 mm length), and filter paper (31 mm length) for absorbing extra liquid. Both the sample pad and gold-labeled pad were immersed in buffer solution containing Tris-HCl (0.1 M, pH 7.5), 1% BSA, 3% sucrose, and 0.5% Tween-20 for 30 min, then placed in oven for drying at 37 °C. In a centrifuge tube, 50 µL of 0.02% colloidal gold solution containing 20 nm gold nanoparticles (Denuojieyi Biotechnology Co., Ltd., Beijing, China) was added, and the pH of the solution was adjusted with 0.2 M K_2_CO_3_. Thereafter, different amounts of rabbit anti-FITC antibody were added, and the mixed solution stood for 15 min at 4 °C, waiting for the gold particles to couple with the primary antibody. To prevent non-specific binding, 5 µL of 10% BSA was added, shaken well, and left for 30 min at 4 °C. The supernatant was removed by centrifugation at a speed of 8000 r/min for 30 min at 4 °C. Then, the precipitation was retained and resuspended in 10 µL of Tris-HCl (0.1 M, pH 7.5) solution with 1% BSA and 3% sucrose. The colloidal gold-labeled anti-FITC antibody solution was evenly sprayed on the gold-labeled pad and then dried at room temperature.

The test line and control line were prepared by the tip of a cotton thread dispensing streptavidin and goat anti-rabbit secondary antibodies onto the nitrocellulose membrane, respectively. The membrane was dried at room temperature for 2 h and stored in a dry state. The four parts of the test strip were sequentially pasted onto plastic adhesive backing in an orderly manner with an overlap of 2 mm to ensure good contact between the parts (Figure 1C). The RT-RAA reaction products absorbed by the test strip flowed from the sample pad to the gold-labeled pad, and then colloidal gold-labeled rabbit anti-FITC antibodies bound to the FITC-tagged amplicons to form complexes. Subsequently, the complexes were captured by streptavidin on the test line, which resulted in a colorimetric readout indicating a positive result, and excess antibody-decorated gold nanoparticles moved across the test line followed by being immobilized on the control line through goat anti-rabbit secondary antibodies, also leading to a red appearance. In the absence of the target gene amplicons, color appeared only on the control line, validating the effectiveness of the test strip. If coloration is observed only on the detection line, the test results are considered invalid (Figure 1D). During the testing process, excess reagents are absorbed by the filter paper on the test strip, thus not causing any additional burden to the environment. All the materials for the test strip were purchased from Bainuodi Biotechnology Co., Ltd. (Weifang, China).

### 2.3. Optimization of Reaction Conditions

To achieve the optimal pH for labeling gold nanoparticles, different volumes (0, 0.5, 1, 1.5, 2.0, and 2.5 µL) of K_2_CO_3_ solution (0.2 M) and excessive rabbit anti-FITC antibody (2 µg) were added into each centrifuge tube containing 50 µL gold nanoparticle solution (0.02%), respectively. To further identify the optimal amount of protein labeling, different concentrations (0, 2, 4, 8, 12, 16, 20, and 30 μg/mL) of rabbit anti-FITC antibody were added into each centrifuge tube containing 50 µL colloidal gold solution (0.02%) at the optimal pH value, respectively. All the solutions were incubated at 4 °C for 30 min before detection under BIBBY Jenway UV-visible spectrophotometer (Stone, Staffs, UK) at 520 nm. The color change in the colloidal gold solution was observed and imaged.

Gold-label pad was prepared based on the optimal pH and primary antibody concentration, and the test strip was combined with the reaction chamber to detect the RT-RAA product. RT-RAA was performed according to the instructions of the RT-RAA kit (Baoyingtonghui Biotechnology Co., Ltd., Beijing, China). Moreover, 25 μL of buffer, 2 μL of upstream and downstream primers (10 nM), 18.5 µL of the clinical sample, and 2.5 μL of magnesium acetate were added into the detecting unit tube. The reaction solution was completely mixed with gentle shaking upside down and transferred into the reaction chamber followed by being incubated under the armpit. The thin PDMS layer is pierced with the test strip at the end of the RT-RAA. Three different parameters for the integrated detection device were further optimized. A single variable was controlled during the optimization process, including the RT-RAA reaction time (10, 20, 30, and 40 min), goat anti-rabbit secondary antibody concentration (0.2, 0.5, 1.0, and 1.5 mg/mL) and streptavidin concentration (0.1, 0.25, 0.5, and 1.0 mg/mL). The grayscale values of each strand on the control line and test line were analyzed using Image J software (ImageJ 1.54f, National Institute of Health, Bathesda, Rockville, MD, USA).

### 2.4. Detection of Clinical Samples

Under the optimal conditions that have been identified according to the optimization experiments mentioned above, 18.5 µL of serum samples from lung cancer patients (*n* = 20) and healthy controls (*n* = 20), as well as primers for CEA, were added into the reaction chamber with the RT-RAA kit, respectively, and the strands on the test strip were imaged, and each strand on test line was subjected to grayscale processing using Image J software. The grayscale intensity values were normalized to the 0–1 range by linearly scaling each pixel value between the minimum and maximum intensities, which were assigned to 0 and 1, respectively. Similar experiments were conducted for urine samples from patients with prostate cancer (*n* = 8) and healthy controls (*n* = 4) to detect both PSA and PCA3.

### 2.5. Statistical Analysis

All reported values were averaged and presented as mean ± standard deviation (SD). Statistical differences between different groups were analyzed by Student’s two-tailed *t*-test, assuming equal variances, and the value of *p* < 0.05 was considered statistically significant.

## 3. Results and Discussion

### 3.1. Optimal Conditions for Colloidal Gold Labeling

**Figure 2:**
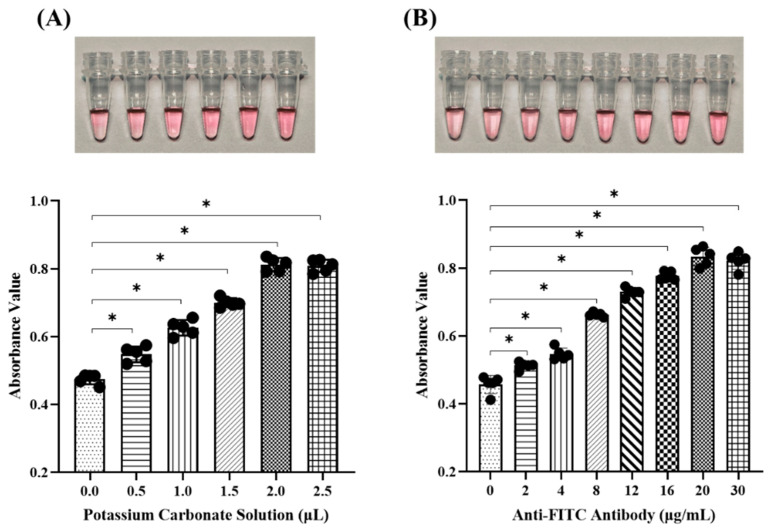
Condition optimization for colloidal gold labeling. (**A**) The color change in colloidal gold solution with different quantities of K_2_CO_3_ and their absorption values at 520 nm. (**B**) The color change in colloidal gold solution with different quantities of anti-FITC antibody and their absorption values at 520 nm. * *p* < 0.05. Black circles represent individual sample measurements. Rectangles with distinct patterns demarcate experimental groups under different parameter conditions.

To identify the optimal parameters for labeling colloidal gold solution, the pH value was firstly optimized by adding different volumes of K_2_CO_3_ solution to 50 μL of commercial colloidal gold solution containing excessive rabbit anti-FITC antibody. Colloidal gold is known to be an acidic solution; thus, antibodies are rejected by colloidal gold in an acidic environment due to their shared polarity. On the contrary, antibodies carrying opposite charges would be attracted to colloidal gold in an alkaline environment [21]. Hence, we selected 0.2 M K_2_CO_3_ solution to adjust the pH of the colloidal gold solution. The results showed that gradual color change in the colloidal gold solution was clearly observed with the addition of different K_2_CO_3_ quantities, indicating that the condensation of gold nanoparticles was inhibited at an elevated pH value (Figure 2A). The absorbance value of the colloidal gold solution at 520 nm increased consistently with the color change and reached the highest point with 2 μL of K_2_CO_3_ supplement, indicating the best homogeneity of the colloidal gold solution at its most stable state (Figure 2A). In order to ensure that the majority of antibodies were successfully labeled by colloidal gold nanoparticles, we selected 2 μL of K_2_CO_3_, which adjusted the pH value of the colloidal gold solution to 8.5, as the labeling condition. Afterward, the optimal amount of rabbit anti-FITC antibody in the colloidal gold solution at the optimal labeling pH was determined. As shown in Figure 2B, the color of the colloidal gold solution exhibited a progressive change with the serially increased quantities of the antibody, and the maximum absorption value was measured when the antibody concentration increased to 20 μg/mL. Thereafter, the color change in the colloidal gold solution was not readily apparent with the continual increment of antibody quantities. These data illustrate that the optimal pH value for labeling colloidal gold solution is 8.5, and the optimal concentration for antibody labeling is 20 μg/mL. Our results are similar to the studies by Hu et al., but we chose to use higher antibody concentrations for better labeling efficiency [22].

### 3.2. Optimal Detection Conditions for Colloidal Gold Test Strips

**Figure 3:**
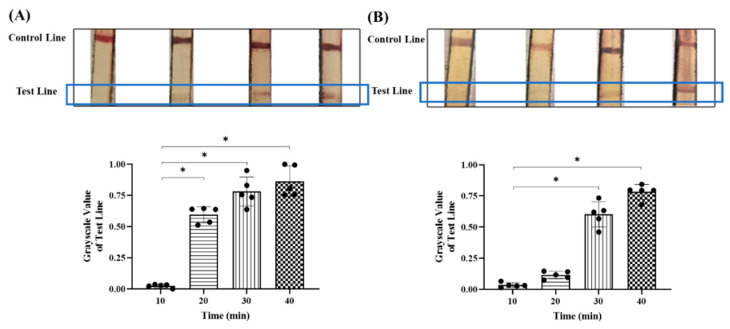
The optimization of the RT-RAA reaction time for the integrated detection platform. (**A**) The band’s appearance on the test strip for serum samples of lung cancer patients and the corresponding grayscale value analysis at different time points. (**B**) The band’s appearance on the test strip for serum samples of healthy individuals and the corresponding grayscale value analysis at different time points. * *p* < 0.05. Black circles represent individual sample measurements. Rectangles with distinct patterns demarcate experimental groups under different parameter conditions.

After the optimal labeling pH value and antibody amount of colloidal gold solution were obtained, the test strip and reaction chamber could be assembled together to constitute the integrated detection device. To acquire accurate detection results from the test strip, the detection conditions for the colloidal gold test strip were further optimized, including the RT-RAA reaction time, secondary antibody concentration, and streptavidin concentration. The optimal parameters were determined based on the color development intensity of the test and control lines. The reason why we chose to perform RT-RAA under body temperature (37 °C) is that it could be accomplished easily under armpit, thereby avoiding the use of laboratory instruments and increasing the likelihood of realizing POCT diagnosis. The results showed that no clear band was visible on the test line when the RT-RAA reaction of serum samples lasted for 10 min, and there was a discernible band on the test line when the reaction extended to 20 min (Figure 3A). However, when the reaction time exceeded 20 min, the test line exhibited a non-reactive false positive band on the nitrocellulose membrane (Figure 3B), which could be attributed to the presence of low levels of CEA in the serum of healthy individuals [23] and the exceptionally high sensitivity of the RT-RAA technique [24]. Hence, the noticeable color divergence on the test line potentially distinguishes serum samples from cancer patients and healthy individuals at the time point of 20 min, with significant differences in grayscale values, and we identified 20 min as the optimal reaction time for RT-RAA to obtain an accurate measurement. Wen et al. also employed RT-RAA combined with the lateral flow dipstick assay to detect swine fever virus at 37 °C. However, they found that no amplification products could be detected at a reaction time of 5 min and no obvious differences in the reaction times from 10 min to 30 min [25]. Different approaches to labeling primers and capturing target amplicons might explain this inconsistency between their results and our findings. Moreover, whether the nucleic acid amplification process requires reverse transcription can also affect the time. Nie et al. found that in the detection of Japanese encephalitis in pigs, amplification curves could be seen after 10 min of the reaction, and the results could be determined after 30 min [26]. However, Wang et al. performed a pre-reverse transcription on the samples to be tested during the detection of sugarcane mosaic virus, and the RAA reaction could yield effective results within 10 min [27]. The above results also demonstrated the feasibility and effectiveness of the integrated device for detecting nucleic acid markers in clinical samples, suggesting that the combination of RT-RAA and the colloidal gold test strip could potentially contribute to cancer diagnosis at the mRNA (Messenger RNA) level, even if their combination has been reported to be applied in the diagnosis of other diseases [18,19,20].

**Figure 4:**
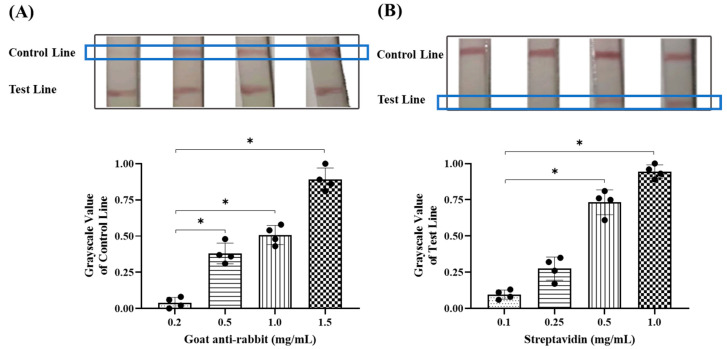
Detection condition optimization for colloidal gold test strips. (**A**) The band’s appearance on the test strip on the control line that is coated with secondary antibodies at different concentrations and the corresponding grayscale value analysis. (**B**) The band’s appearance on the test strip on the test line that is coated with streptavidin at different concentrations and the corresponding grayscale value analysis. * *p* < 0.05. Black circles represent individual sample measurements. Rectangles with distinct patterns demarcate experimental groups under different parameter conditions.

The secondary antibodies that were coated on the control line specifically bound to extra gold-labeled anti-FITC antibodies that were transported in the direction of the conjugate pad by the diluent to verify the validity of the test strip, so their concentrations significantly influenced the intensity of the control line. Secondary antibodies were diluted to concentrations of 0.2, 0.5, 1.0, and 1.5 mg/mL and coated onto nitrocellulose membranes. As illustrated in Figure 4A, a slight strand appears on the control line at a concentration of 0.2 mg/mL. The control line gradually darkens with increasing concentrations of secondary antibodies, and a significant band is evident at a concentration of 1.0 mg/mL. The strand shows the strongest grayscale intensity at a concentration of 1.5 mg/mL due to the high coated concentration. To minimize the consumption of antibodies, we select 1.0 mg/mL of the secondary antibody to coat the control line in the subsequent experiments.

Streptavidin, which is coated on the test line, is responsible for monitoring target nucleic acid due to its high affinity to biotin, which is carried by RT-RAA products. Thus, the intensity of the test line is directly determined by streptavidin concentration. Although the biotin–streptavidin system has been extensively utilized in the fabrication of immunochromatographic test strips, their optimal concentrations in specific test strips still need to be explored [28]. The data showed that no apparent band was seen on the test line by the unaided eye at 0.1 mg/mL of streptavidin, suggesting it failed to capture the biotin-labeled RT-RAA product, and even at a concentration of 0.25 mg/mL, the test line was difficult to observe. However, a significant band was evident at the test line when the streptavidin concentration was raised to 0.5 mg/mL, and the grayscale value dramatically increased with elevated streptavidin concentration (Figure 4B). These data support the utilization of 0.5 mg/mL as the streptavidin concentration in the following experiments.

### 3.3. The Detection Results of Different Cancer Nucleic Markers in Clinical Samples Through the Integrated Device

**Figure 5:**
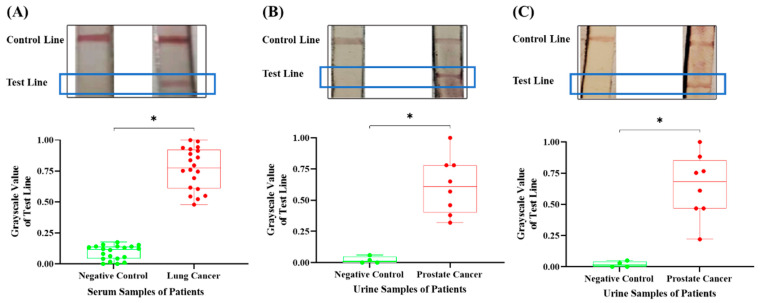
Detection results of the integrated device in assessing clinical samples for cancers. (**A**) The band appearance on the test strip for detecting CEA in serum samples and corresponding grayscale value analysis. (**B**) The band appearance on the test strip for detecting PSA in urinary samples and corresponding grayscale value analysis. (**C**) The band appearance on the test strip for detecting PCA3 in urinary samples and corresponding grayscale value analysis. * *p* < 0.05. Circles and lines of different colors represent different groups.

After the optimal detection conditions were successfully identified through the aforementioned experiments, the designed and fabricated integrated detection device was further employed to test clinical samples from both healthy individuals and cancer patients. CEA is a broad-spectrum biomarker for multiple cancers, such as lung, colorectal, gastric, and breast cancer, and so on [29]. Over the past years, various novel analytical approaches and advanced biosensors have been developed to detect CEA, owing to its significance in cancer diagnosis, even though the current clinical testing for CEA still depends on the classical enzyme-linked immuno-sorbent assay (ELISA) [30]. In the present study, the integrated device detected CEA in 20 serum samples from lung cancer patients, and the control serum samples from healthy individuals were also evaluated following the same experimental procedure. As shown in Figure 5A, both 20 positive samples and 20 negative samples were accurately distinguished by the completely different band appearance on the test strips. There was a significant difference in the grayscale values of the detection lines between the two groups, indicating that the detection results obtained from the integrated device were consistent with the clinical identification. These data not only underscore the significance of CEA as a clinical diagnostic biomarker for cancers but also support the fact that this new detection platform exhibited excellent clinical performance.

Compared to serum samples, urine samples can potentially be applied in POCT environments, owing to their availability and accessibility. Urinary antigens have been widely used for bacterial infection and urinary system disease diagnosis [31,32]. Although PSA testing has played a vital role in the decrease in prostate cancer mortality, its low specificity usually leads to misdiagnosis due to the relevance of PSA to other prostate diseases. PCA3, which is usually overexpressed in prostate cancer and almost absent in non-neoplastic tissue, could improve the specificity and accuracy of prostate cancer diagnosis based on PSA [31]. Herein, the integrated device was used to assess the mRNA levels of both PSA and PCA3 in urinary samples obtained from eight patients diagnosed with prostate cancer and healthy individuals [33]. The findings indicated that all eight positive samples exhibited a detectable band on the test line, while no visible band occurred on the test line for all eight negative samples (Figure 5B,C). Thus, the grayscale values of test lines from cancer patients were significantly higher than those of the test lines from healthy individuals, suggesting the integrated device provided satisfactory diagnostic efficacy and high sensitivity as well as specificity for urine-derived nucleic acid biomarkers. In addition, these findings provided additional evidence supporting the applicability and feasibility of PSA and PCA3 as potential biomarkers for prostate cancer diagnosis.

More importantly, the integrated detection device detected neither false positive nor false negative cases during the nucleic acid marker analysis from the clinical samples. Therefore, the findings suggested that the integrated detection device demonstrated superior diagnostic performance.

## 4. Conclusions

In summary, this study successfully developed a rapid, reliable, and user-friendly platform for the detection of cancer nucleic acid biomarkers in clinical samples, demonstrating excellent specificity and sensitivity. By integrating reverse-transcription recombinase-aided amplification (RT-RAA) technology with colloidal gold test strips, this innovative approach offers a visual and convenient alternative to conventional PCR-based diagnostic methods. The platform’s ability to operate at body temperature without the need for sophisticated laboratory equipment significantly enhances its potential for point-of-care testing (POCT) and self-screening applications. The optimized conditions for the RT-RAA reaction time, antibody concentration, and streptavidin concentration further ensure the accuracy and reliability of the detection results. This integrated device not only simplifies the diagnostic process but also reduces the dependency on specialized personnel and expensive instruments, making it particularly suitable for resource-limited settings.

Moreover, this platform, capable of detecting a single nucleic acid biomarker (e.g., CEA, PSA, or PCA3) in serum and urine samples, underscores its potential as a valuable tool for early cancer screening. The absence of false positives or false negatives in the clinical sample analysis further validates the robustness of this diagnostic approach. Future research could explore the integration of additional biomarkers and the adaptation of this platform for other diseases, thereby broadening its diagnostic scope. Overall, this study provides a significant step forward in the development of accessible and efficient cancer diagnostic tools, with the potential to improve early detection rates and patient outcomes.

## Figures and Tables

**Table 1:** The sequences of RT-RAA primers.

Gene Primers	Sequence (5′→3′)
CEA-F	Biotin-CAAACCGCAGTGACCCAGT
CEA-R	FITC-ACTCCAATCATGATGCCGACAG
PSA-F	Biotin-TTTCCTTATCATCCTCGCTCCTC
PSA-R	FITC-CATGACCTTCACAGCATCCGT
PCA3-F	Biotin-GAAGCACCTCGCATTTGTGG
PCA3-R	FITC-GGCCAGAAGCTAGCATCCAT

## Data Availability

The original contributions presented in the study are included in the article, further inquiries can be directed to the corresponding author.
